# FOSTERING DEMENTIA INCLUSIVE NEIGHBORHOODS: A GIS ANALYSIS OF NEIGHBORHOOD WALKABILITY FOR PERSONS WITH DEMENTIA

**DOI:** 10.1093/geroni/igae098.1336

**Published:** 2024-12-31

**Authors:** Shannon Freeman, Keone Gourley, Emma Rossnagel, Mark Groulx

**Affiliations:** University of Northern British Columbia, Prince George, British Columbia, Canada; University of Northern British Columbia, Prince George, British Columbia, Canada; Centre for Technology Adoption for Aging in the North, Prince George, British Columbia, Canada; University of Northern British Columbia, Prince George, British Columbia, Canada

## Abstract

Active engagement in the natural environment is an important aspect of health and wellbeing for persons with dementia. Neighborhoods with high walkability are critical for persons with dementia to access the outdoors and promote ageing in place, while allowing for persons with dementia to maintain social relationships, connect with nature, and foster a sense of freedom. Although persons living with dementia are affected by general walkability factors such as presence of sidewalks and the availability of destinations such as health services or recreational facilities, dementia-inclusive neighborhoods must also be distinctive and easy to navigate, as persons with dementia are more prone to become disoriented or lost. Therefore, the Dementia-Inclusive Streets and Community Access, Participation and Engagement (DemSCAPE) project sought to identify how persons living with dementia interact with their environment and how municipalities can implement change to make cities more walkable for PLWD. A GIS analysis was completed to assess current infrastructural barriers and facilitators for aging-in-place for persons living with dementia. This analysis, which included interviews with individuals living with dementia and their family caregivers, found that their neighborhoods were severely lacking the infrastructure needed to support walkability for persons living with dementia. From the absence of sidewalks to disorienting neighborhood patterns, the city of focus has a long way to go in supporting aging-in-place for persons living with dementia. This presentation will showcase how these findings can be used to influence protocols in municipalities to implement supportive infrastructure for persons living with dementia.

